# Whole Blood Gene Expression and Atrial Fibrillation: The Framingham Heart Study

**DOI:** 10.1371/journal.pone.0096794

**Published:** 2014-05-07

**Authors:** Honghuang Lin, Xiaoyan Yin, Kathryn L. Lunetta, Josée Dupuis, David D. McManus, Steven A. Lubitz, Jared W. Magnani, Roby Joehanes, Peter J. Munson, Martin G. Larson, Daniel Levy, Patrick T. Ellinor, Emelia J. Benjamin

**Affiliations:** 1 Section of Computational Biomedicine, Department of Medicine, Boston University School of Medicine, Boston, Massachusetts, United States of America; 2 National Heart Lung and Blood Institute’s and Boston University’s Framingham Heart Study, Framingham, Massachusetts, United States of America; 3 Department of Biostatistics, Boston University School of Public Health, Boston, Massachusetts, United States of America; 4 Cardiology Division, Department of Medicine, and Epidemiology Division, Department of Quantitative Health Sciences, University of Massachusetts Medical School, Worcester, Massachusetts, United States of America; 5 Cardiovascular Research Center, Massachusetts General Hospital, Charlestown, Massachusetts, United States of America; 6 Section of Cardiovascular Medicine, Department of Medicine, Boston University School of Medicine, Boston, Massachusetts, United States of America; 7 Mathematical and Statistical Computing Laboratory, Center for Information Technology, National Institutes of Health, Bethesda, Maryland, United States of America; 8 Population Sciences Branch, Division of Intramural Research, National Heart, Lung, and Blood Institute, Bethesda, Maryland, United States of America; 9 Department of Mathematics and Statistics, Boston University, Boston, Massachusetts, United States of America; 10 Center for Human Genetic Research, Massachusetts General Hospital, Boston, Massachusetts, United States of America; 11 Cardiac Arrhythmia Service, Massachusetts General Hospital, Boston, Massachusetts, United States of America; 12 Harvard Medical School, Boston, Massachusetts, United States of America; 13 Section of Preventive Medicine, Department of Medicine, Boston University School of Medicine, Boston, Massachusetts, United States of America; 14 Department of Epidemiology, Boston University School of Public Health, Boston, Massachusetts, United States of America; Medical University Hamburg, University Heart Center, Germany

## Abstract

**Background:**

Atrial fibrillation (AF) involves substantial electrophysiological, structural and contractile remodeling. We hypothesize that characterizing gene expression might uncover important pathways related to AF.

**Methods and Results:**

We performed genome-wide whole blood transcriptomic profiling (Affymetrix Human Exon 1.0 ST Array) of 2446 participants (mean age 66**±**9 years, 55% women) from the Offspring cohort of Framingham Heart Study. The study included 177 participants with prevalent AF, 143 with incident AF during up to 7 years follow up, and 2126 participants with no AF. We identified seven genes statistically significantly up-regulated with prevalent AF. The most significant gene, *PBX1* (*P* = 2.8×10^−7^), plays an important role in cardiovascular development. We integrated differential gene expression with gene-gene interaction information to identify several signaling pathways possibly involved in AF-related transcriptional regulation. We did not detect any statistically significant transcriptomic associations with incident AF.

**Conclusion:**

We examined associations of gene expression with AF in a large community-based cohort. Our study revealed several genes and signaling pathways that are potentially involved in AF-related transcriptional regulation.

## Introduction

Atrial fibrillation (AF), the most common cardiac arrhythmia, is characterized by electrophysiological, structural and contractile remodeling typically in the left atrium [1]–[4]. Several disease pathways might predispose to AF [5]–[7], and heritability plays a role in its development [8]–[11]. Recently, genome-wide association studies (GWAS) identified several genetic loci associated with AF [12]–[14]. However, the mechanisms underlying the genetic predisposition to AF susceptibility remain unknown.

Gene expression is a quantitative and heritable phenotype mediated by the interplay of environmental and genetic factors [15], [16]. Both human and animal models indicate that AF has profound effects on global gene expression [17]–[19]. Many of the differentially expressed genes were involved in ion channels, which play an essential role in the pathophysiology of AF initiation, perpetuation and adaptation [19]–[22]. Expression of extracellular signal-regulated kinases and angiotensin-converting enzyme were increased in AF [23]. In addition, differential gene expression was observed in patients with heart failure [24] and valvular heart disease [25].

Identification of gene expression signatures may provide insights into AF pathogenesis and suggest targets for therapeutic intervention. Prior transcriptomic studies were generally limited to small groups of selected samples, which might not represent AF in the community. We aimed to investigate associations of AF with gene expression in whole blood using a large community-based cohort. We also investigated interactions between genes differentially expressed in AF patients.

## Methods

### Study Samples

The Framingham Heart Study is a community-based observational cohort designed to investigate cardiovascular disease and its risk factors. Since the study was initiated in 1948, three generations of participants have been enrolled. A total of 5124 participants were enrolled in 1971 in the Offspring Cohort. It consists of children of the Original Cohort and their spouses [26]. Every 4–8 years, Offspring participants complete a standardized history and 12-lead ECG, blood tests and noninvasive tests. For the present study, we focused on 2446 Offspring participants who had RNA collected at the eighth examination (2005–2008). All participants gave written informed consent. Our study was approved by the Institutional Review Boards of National Human Genome Research Institute and Boston University Medical Center.

### AF Ascertainment

At each examination, participants were asked about AF status. In addition, their AF status and cardiovascular history were solicited during surveillance interviews biennially [27], [28]. We included multiple sources to determine the presence of AF, such as a 12-lead electrocardiogram from Framingham Heart Study examination, and all cardiovascular disease-related hospitalizations and clinician visits. All electrocardiograms available from study visits or in- and outpatient records were reviewed by study cardiologists to adjudicate AF and atrial flutter events.

### Gene Expression Profiling

The gene expression profiling has been described in detail by Joehanes et al [29]. In brief, fasting peripheral whole blood was collected during clinical visits. Total RNA was isolated from frozen PAXgene blood tubes (PreAnalytiX, Hombrechtikon, Switzerland) and amplified using the WT-Ovation Pico RNA Amplification System (NuGEN, San Carlos, CA) according to the manufacturers’ standard operating procedures. The obtained cDNA was hybridized to the Human Exon 1.0 ST Array (Affymetrix, Inc., Santa Clara, CA). The raw data were quantile-normalized and log2 transformed, followed by summarization using Robust Multi-array Average [30]. The data were further adjusted for batch effects and technical covariates, including the first principle component of the expression data, batch effect, and the all-probeset-mean residual. We also adjusted gene expression for the complete blood counts, which were imputed from more than 2000 samples from the Third Generation cohort as previously described [29]. We also measured the RNA Integrity Number in almost all samples using an automated method (Asuragen Inc, Austin, TX), and tested the inclusion of the RNA Integrity Number and RNA-related measures as technical covariates, but did not find them statistically significant. We therefore did not include them in our models for this study. Our analysis focused on the “core” probe sets, which were defined as the most reliable probe sets derived from RefSeq and GenBank records. The gene and exon annotations were obtained from Affymetrix NetAffx Analysis Center (version 31). We excluded exons with signals lower than the background, and transcript clusters that were not mapped to RefSeq transcripts. We studied 287,329 probesets, corresponding to 209,699 exons and 17,873 distinct transcripts for downstream analysis.

### Statistical Analyses

Our primary analysis used prevalent AF cases – AF diagnosed before the blood sample for RNA was drawn. The association between gene expression and AF was assessed by linear mixed effect models to account for familial correlation between Framingham Heart Study Offspring participants. We regressed gene expression on AF status, and adjusted for age and sex. Significant hits were adjusted for additional AF risk factors [31], including smoking, height, weight, systolic blood pressure, diastolic blood pressure, prevalent diabetes mellitus, prevalent myocardial infarction, prevalent heart failure and antihypertensive treatment. In secondary analyses, we studied associations of gene expression with incident AF using used Cox proportional hazards models with robust sandwich estimators and clustering by pedigree, censoring at the last follow-up time or death. We also examined the correlation of gene expression with various medications, including warfarin, beta blocker, digoxin, and calcium channel blocker. The gene expression was treated as the outcome, whereas the usage of medication was treated as dichotomous independent variables. To adjust for multiple testing, false discovery rate (FDR) [32] was used, and statistical significance was claimed if FDR was less than 0.05. All analyses were performed using R software package (www.r-project.org/).

### Construction of Interaction Subnetwork Associated with AF

We applied a dense module searching strategy [33] to identify modules enriched with AF-related genes. The protein-protein interaction data were obtained from the PINA database [34], which was compiled from multiple protein-protein interaction databases. All genes were included into analyses and assigned as the seed gene once. Briefly, each gene was assigned a score to represent its association with AF. The module searching started from a single seed gene. Neighboring genes were added sequentially to the module if the adding increased the overall module score [35], which was defined as 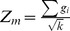, where k was the number of genes in the module, and 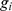 was the score of gene i. The searching stopped if no genes could be added. Ingenuity Pathway Analysis tool (Ingenuity Systems, Redwood, CA) was then used to examine the enrichment of all module genes in each canonical pathway, and the significance was claimed if its FDR [32] was less than 0.05.

## Results

### Differential Gene Expression

**Figure 1:**
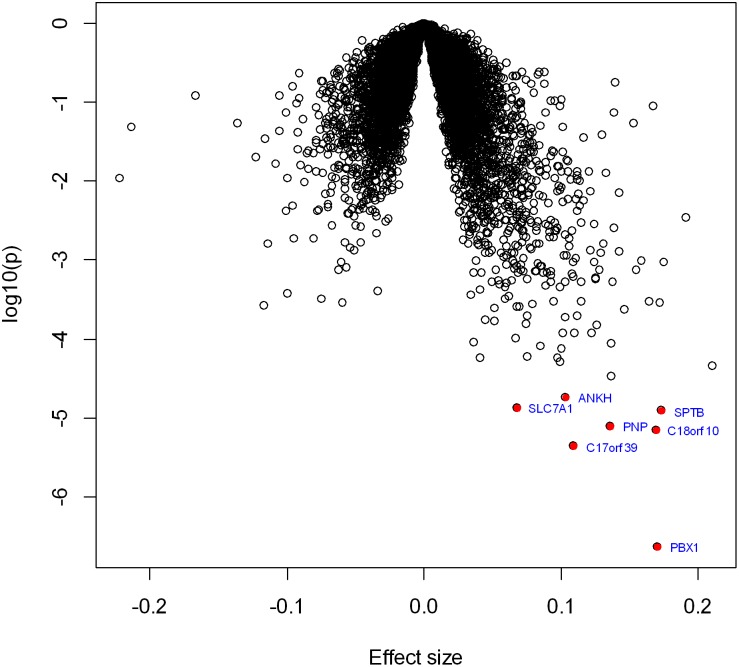
Volcano plot of expression association with prevalent AF. Each dot represents one gene. The x-axis represents the effect of each gene, whereas the y-axis represents the log_10_ (*P* value). Positive effects represent that the genes were over-expressed in prevalent AF, while negative effects represent that the genes had decreased expression with prevalent AF. Seven genes that reached the significant cutoff (FDR<0.05) were marked.

Descriptive characteristics for 2446 eligible participants (mean age 66**±**9 years, 55% women) are provided in **Table 1**. We observed 177 prevalent cases who developed AF before examination 8 when their blood was collected for RNA extraction (prevalent AF), and 143 additional AF cases who developed AF after their RNA was collected (incident AF). The reference group consisted of 2126 participants who had not developed AF by the last follow-up in 2012. **Figure 1** is the volcano plot showing the association of all the studied genes with prevalent AF. As listed in **Table 2**, with FDR<0.05, seven genes were significantly associated with prevalent AF; all were over-expressed in prevalent AF. The most significant gene, *PBX1* (*P* = 2.8×10^−7^, FDR = 0.005), encodes a transcriptional factor belonging to the PBX homeobox family. The most significant decreased expression gene was *REL* (*P* = 2.6×10^−4^, FDR = 0.14), which did not reach the significance cutoff. We then tested the association of these genes with prevalent AF adjusting for additional AF risk factors [31], including smoking, height, weight, systolic blood pressure, diastolic blood pressure, prevalent diabetes mellitus, prevalent myocardial infarction, prevalent heart failure and antihypertensive treatment. As shown in **Table S1 in File S1**, the associations of these genes were attenuated, but they still reached the nominal significance cutoff (*P*<0.05).

**Table 1:** Clinical characteristics of the sample.

Characteristics	No AF (n = 2,126)	Prevalent AF (n = 177)	Incident AF (n = 143)
Women, n (%)	1224 (58%)	63 (36%)	56 (39%)
Age, year ± SD	66±9	73±8	71±8
Smoker, n (%)	186 (9%)	7 (4%)	11 (8%)
Height, inches ± SD	66±4	67±4	66±4
Weight, pound ± SD	174±39	186±42	181±41
Systolic blood pressure, mm Hg	128±17	128±20	137±19
Diastolic blood pressure, mm Hg	74±10	69±11	72±10
Prevalent diabetes mellitus, n (%)	317 (15%)	56 (32%)	40 (28%)
Prevalent myocardial infarction, n (%)	22 (1%)	41 (23%)	7 (5%)
Prevalent heart failure, n (%)	67 (3%)	45 (25%)	9 (6%)
Antihypertensive treatment, n (%)	971 (46%)	129 (73%)	94 (66%)

**Table 2:** Most significant transcripts associated with prevalent AF (FDR<0.05).

Transcript ID	Gene Symbol	Average Expression		Effect size	SE*	*P* value	FDR*
		AF (n = 177)	No AF (n = 2269)				
2364677	*PBX1*	6.85	6.71	0.17	0.03	2.8×10^−7^	0.005
3712922	*C17orf39*	5.68	5.56	0.11	0.02	6.5×10^−6^	0.037
3527514	*PNP*	7.22	7.11	0.14	0.03	7.1×10^−6^	0.037
3804358	*C18orf10*	7.51	7.37	0.17	0.04	8.2×10^−6^	0.037
3507710	*SLC7A1*	5.65	5.58	0.07	0.02	1.3×10^−5^	0.046
3568534	*SPTB*	5.92	5.76	0.17	0.04	1.6×10^−5^	0.047
2849469	*ANKH*	7.34	7.26	0.10	0.02	1.9×10^−5^	0.048

*SE: Standard error; FDR: false discovery rate [32].

In our secondary analysis, we performed the association of gene expression with incident AF adjusted for age and sex. No genes reached the significance cutoff (FDR<0.05) (**Table S2 in File S1**). The most significant gene was *GPCPD1* (*P* = 1.5×10^−5^, FDR = 0.27). The volcano plot of gene expression with incident AF is shown in **Figure S1 in File S1**.

Since Affymetrix Exon ST1.0 Array is able to profile transcriptomic signatures at the exon level, we also studied the association of each exon expression with prevalent AF. **Table S3 in File S1** shows the top prevalent AF-related exons, and the **Table S4 in File S1** shows the association of each exon within the seven AF-related genes. Interestingly, the seventh exon of *PBX1* was also the most significant exon associated with prevalent AF (*P* = 3.5×10^−7^), suggesting that the exon has the most significant differential expression between prevalent AF cases and controls.

We also performed additional analyses to study the correlation of gene expression with various medications, including warfarin, beta blocker, digoxin, and calcium channel blocker. We found that warfarin, beta blocker, digoxin, and calcium channel blocker have 3, 287, 0, and 166 significant genes respectively (FDR<0.05); the most significant genes for each medication are shown in the **Table S5 in File S1**.

In order to further characterize transcriptomic signatures associated with prevalent AF, we stratified samples with prevalent AF based on their status during the blood draw: sinus rhythm (n = 115) and AF (n = 62). As shown in the **Table S6 in File S1**, all seven prevalent AF-related genes were associated with both sinus rhythm and AF (nominal *P*<0.05), although the association was attenuated due to the reduced sample size and limited power.

### Gene Interaction Network Associated with AF

**Figure 2:**
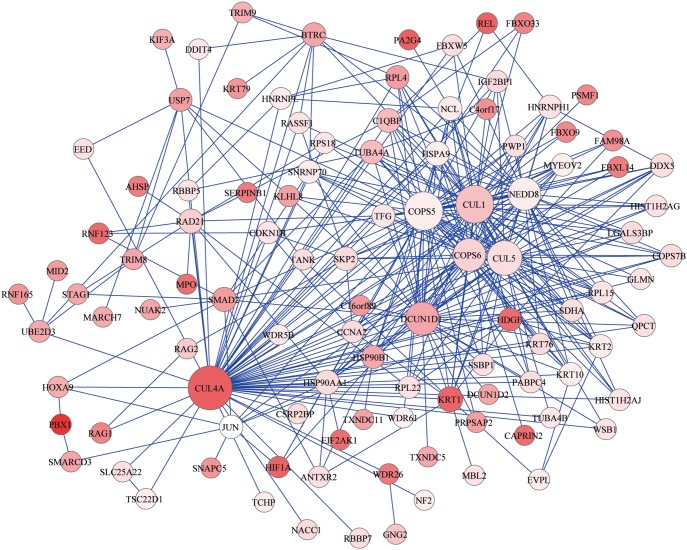
AF subnetwork derived from protein-protein interaction. Each node represents one gene, wheras each edge represents the interaction between two genes. The nodes were colored to represent their association with AF: red color represents strong association, and white color represents no association. The node size is proportional to the number of edges that the node connects to.

We applied a dense module searching strategy [33] to identify modules containing genes with strong association with AF. Each module represents a group of interacting genes; the nodes represent genes, and the edges represent interactions between genes. A total of 7993 modules were identified. Given the highly overlapping nature of modules and the enrichment of significant modules, we merged the top 1% modules with highest scores as previously suggested [33] and considered it as an AF subnetwork (**Figure 2**). The subnetwork consisted of 106 nodes and 360 edges. The most significant gene, *PBX1*, was included in the subnetwork.

In order to further characterize the function of AF subnetwork, we applied Ingenuity Pathway Analysis to study the gene enrichment in canonical pathways. Twenty-two canonical pathways were significantly enriched with genes in the AF subnetwork (FDR<0.05). The most significant pathways were antiproliferative role of transducer of ERBB2 (TOB) in T Cell Signaling (FDR = 3.5×10^−9^) and hypoxia signaling in the cardiovascular system (FDR = 1.1×10^−6^) (**Table 3**).

**Table 3:** Most significant canonical pathways enriched with genes in the AF subnetwork (FDR<0.005).

Canonoical pathway	FDR	Ratio+	Genes in the AF subnetwork
Antiproliferative Role of TOB in T Cell Signaling	3.5×10^−9^	6/26 (0.23)	*SMAD2, CCNA2, PABPC4, CUL1, CDKN1B, SKP2*
Hypoxia Signaling in the Cardiovascular System	1.1×10^−6^	6/67 (0.09)	*HSP90B1, JUN, COPS5, HSP90AA1, HIF1A, UBE2D3*
Cyclins and Cell Cycle Regulation	3.6×10^−6^	6/90 (0.07)	*CCNA2, PA2G4, CUL1, BTRC, CDKN1B, SKP2*
Cell Cycle: G1/S Checkpoint Regulation	1.7×10^−5^	5/67 (0.07)	*PA2G4, CUL1, BTRC, CDKN1B, SKP2*
Protein Ubiquitination Pathway	5.4×10^−5^	8/264 (0.03)	*USP7, HSP90B1, HSPA9, CUL1, HSP90AA1, BTRC, UBE2D3, SKP2*
Aryl Hydrocarbon Receptor Signaling	8.7×10^−5^	6/148 (0.04)	*CCNA2, HSP90B1, JUN, NEDD8, HSP90AA1, CDKN1B*
Estrogen-mediated S-phase Entry	2.5×10^−4^	3/27 (0.11)	*CCNA2, CDKN1B, SKP2*
Prostate Cancer Signaling	9.1×10^−4^	4/94 (0.04)	*HSP90B1, PA2G4, HSP90AA1, CDKN1B*
Cell Cycle: G2/M DNA Damage Checkpoint Regulation	1.5×10^−3^	3/49 (0.06)	*CUL1, BTRC, SKP2*
HIF1α Signaling	2.0×10^−3^	4/105 (0.04)	*JUN, COPS5, HSP90AA1, HIF1A*
Glucocorticoid Receptor Signaling	2.3×10^−3^	6/280 (0.02)	*SMAD2, HSP90B1, JUN, HSPA9, PBX1, HSP90AA1*
EIF2 Signaling	2.6×10^−3^	5/192 (0.03)	*RPL15, EIF2AK1, RPL4, RPL22, RPS18*
eNOS Signaling	4.8×10^−3^	4/136 (0.03)	*CCNA2, HSP90B1, HSPA9, HSP90AA1*

+ Ratio is the number of genes within AF subnetwork comparing to the total number of genes in the pathway.

## Discussion

Gene expression is considered an important bridge to connect genetic variations and diseases [15], [36]. We performed genome-wide whole blood transcriptomic profiling of 177 participants with prevalent AF and more than 2000 AF-free participants from the Framingham Heart Study Offspring cohort. Seven genes were up-regulated in samples with prevalent AF after adjustment for multiple testing, but none of them were reported in prior studies [17]–[23], [37], suggesting the complexity of gene regulation network underlying AF. We did not identify any transcriptomic signatures significantly associated with incident AF.

The most significant gene was *PBX1*, which encodes a homeodomain transcription factor. The gene plays an important role in cardiovascular development, and orchestrates separate transcriptional pathways to control great-artery patterning and cardiac outflow tract septation [38]. The inactivation of *PBX1* results in persistent truncus arteriosus in mice [39]. Another significant gene, *SLC7A1*, encodes a high affinity cationic amino acid transporter that participates in the total carrier-mediated uptake activity [40]. A polymorphism in the 3′-UTR of *SLC7A1* was found to be associated with hypertension and endothelial dysfunction [41], probably due to the binding of miR-122 to the region [42].

Beyond the individual gene association, we also studied the combined association of differential gene expression with AF. Increasing evidence has suggested that the development of AF might result from the disruption of multiple biological pathways [5]–[7], [43]. We constructed an AF subnetwork by taking advantage of a wealth of protein-protein interaction information that was collected in several databases over years. The subnetwork was highly enriched with genes involved in multiple signaling pathways, including hypoxia signaling pathway in the cardiovascular system and the TOB antiproliferative signaling pathway. The hypoxia signaling pathway is key to a series of molecular events to response to the local oxygen tension in the cardiovascular system [44], [45]. The pathway is also involved in the myocardial remodeling [46] and the activation of angiogenic growth factors [47]. The TOB antiproliferative signaling pathway regulates T cell proliferation and cytokine transcription, which could avoid excessive T cell response and thus reduce the risk of autoimmune disorders or tissue injury [48]. Multiple genes in the pathways are involved in attenuated cardiomyocyte hypertrophy and congenital heart diseases [49], [50]. Our analyses provide an approach to translate individual gene associations into a broader picture of transcriptomic profiles, which might be associated with the prevalence or onset of AF.

There are several limitations in our study. We found no significant transcriptomic signatures associated with incident AF, although the sample size was similar to that of prevalent AF. The mechanisms contributing to the discrepancy are unclear. We speculate that the discrepancy may be due to different signaling pathways underlying the onset and perpetuation of AF: genes associated with incident AF might be responsible for the initiation of AF, whereas genes associated with prevalent AF might be responsible for the maintenance of AF. Given that RNA was only collected at the eighth examination of the Offspring cohort but not any earlier examinations, it is infeasible to examine the longitudinal changes of transcriptomic signatures for the same participants. Gene expression varies in different tissue types. The transcriptional profiles in this study were measured on whole blood, but the ideal tissue for study AF is the left atrium. However, invasive left atrial specimen collection is unfeasible in a community-based study, and would result in highly selected samples [51] and few controls. In addition, the cross-hybridization in microarray based platforms might result in non-specific signals for some genes, thus a further validation by RT-PCR or RNA sequencing would be helpful to rule out potential false associations [52], [53]. As a proof-of-concept, we performed RT-PCR on 95 coronary heart disease-related genes in 429 samples selected for a pilot study [29]. As shown in the **Table S7 in File S1**, the expression of 49 out of 95 genes was correlated well between microarray and RT-PCR (Pearson’s correlation coefficient >0.5), and 91 genes have the same direction of effect, suggesting robustness of our assay, although it is not a direct validation of top hits for AF. Given that thousands of tests were conducted and the lack of external replications, it is possible that some findings might be false positives even if we used very stringent Bonferroni correction. Because of the observational study design, the associations we observed do not prove causation. Moreover, the participants were middle-age to older adults, largely of European descent, raising uncertainty regarding generalizability to other ethnicities/races. Our current study did not distinguish between atrial fibrillation and atrial flutter, nor patterns of AF, which might have unique gene expression profiles. Future analyses with stratification of rhythm types might uncover unique gene expression profiles specific to each rhythm type.

In conclusion, we examined the association of whole blood gene expression with AF in a large community-based cohort. Our study revealed that several genes and signaling pathways might be activated in prevalent AF and may be involved in AF maintenance. Future increase of sample size, longer follow up, and external validation would help to identify additional transcription signatures associated with AF and might provide valuable insights into AF pathogenesis [54].

## Supporting Information

File S1 **Supplemental Materials.**
(PDF)

## References

[1] Miyasaka Y, Barnes ME, Gersh BJ, Cha SS, Bailey KR, et al (2006) Secular trends in incidence of atrial fibrillation in Olmsted County, Minnesota, 1980 to 2000, and implications on the projections for future prevalence. Circulation 114: 119–125. 16818816 10.1161/CIRCULATIONAHA.105.595140

[2] Go AS, Hylek EM, Phillips KA, Chang Y, Henault LE, et al (2001) Prevalence of diagnosed atrial fibrillation in adults: national implications for rhythm management and stroke prevention: the AnTicoagulation and Risk Factors in Atrial Fibrillation (ATRIA) Study. JAMA 285: 2370–2375. 11343485 10.1001/jama.285.18.2370

[3] Allessie M, Ausma J, Schotten U (2002) Electrical, contractile and structural remodeling during atrial fibrillation. Cardiovasc Res 54: 230–246. 12062329 10.1016/s0008-6363(02)00258-4

[4] Morillo CA, Klein GJ, Jones DL, Guiraudon CM (1995) Chronic rapid atrial pacing. Structural, functional, and electrophysiological characteristics of a new model of sustained atrial fibrillation. Circulation 91: 1588–1595. 7867201 10.1161/01.cir.91.5.1588

[5] Darbar D, Motsinger AA, Ritchie MD, Gainer JV, Roden DM (2007) Polymorphism modulates symptomatic response to antiarrhythmic drug therapy in patients with lone atrial fibrillation. Heart Rhythm 4: 743–749. 17556195 10.1016/j.hrthm.2007.02.006 PMC1948880

[6] Carnes CA, Chung MK, Nakayama T, Nakayama H, Baliga RS, et al (2001) Ascorbate attenuates atrial pacing-induced peroxynitrite formation and electrical remodeling and decreases the incidence of postoperative atrial fibrillation. Circ Res 89: E32–38. 11557745 10.1161/hh1801.097644

[7] Dernellis J, Panaretou M (2001) C-reactive protein and paroxysmal atrial fibrillation: evidence of the implication of an inflammatory process in paroxysmal atrial fibrillation. Acta Cardiol 56: 375–380. 11791805 10.2143/AC.56.6.2005701

[8] Fox CS, Parise H, D’Agostino RB Sr, Lloyd-Jones DM, Vasan RS, et al (2004) Parental atrial fibrillation as a risk factor for atrial fibrillation in offspring. JAMA 291: 2851–2855. 15199036 10.1001/jama.291.23.2851

[9] Arnar DO, Thorvaldsson S, Manolio TA, Thorgeirsson G, Kristjansson K, et al (2006) Familial aggregation of atrial fibrillation in Iceland. Eur Heart J 27: 708–712. 16428254 10.1093/eurheartj/ehi727

[10] Ellinor PT, Yoerger DM, Ruskin JN, MacRae CA (2005) Familial aggregation in lone atrial fibrillation. Hum Genet 118: 179–184. 16133178 10.1007/s00439-005-0034-8

[11] Darbar D, Herron KJ, Ballew JD, Jahangir A, Gersh BJ, et al (2003) Familial atrial fibrillation is a genetically heterogeneous disorder. J Am Coll Cardiol 41: 2185–2192. 12821245 10.1016/s0735-1097(03)00465-0

[12] Gudbjartsson DF, Arnar DO, Helgadottir A, Gretarsdottir S, Holm H, et al (2007) Variants conferring risk of atrial fibrillation on chromosome 4q25. Nature 448: 353–357. 17603472 10.1038/nature06007

[13] Benjamin EJ, Rice KM, Arking DE, Pfeufer A, van Noord C, et al (2009) Variants in ZFHX3 are associated with atrial fibrillation in individuals of European ancestry. Nat Genet 41: 879–881. 19597492 10.1038/ng.416 PMC2761746

[14] Ellinor PT, Lunetta KL, Albert CM, Glazer NL, Ritchie MD, et al (2012) Meta-analysis identifies six new susceptibility loci for atrial fibrillation. Nat Genet 44: 670–675. 22544366 10.1038/ng.2261 PMC3366038

[15] Emilsson V, Thorleifsson G, Zhang B, Leonardson AS, Zink F, et al (2008) Genetics of gene expression and its effect on disease. Nature 452: 423–428. 18344981 10.1038/nature06758

[16] Dixon AL, Liang L, Moffatt MF, Chen W, Heath S, et al (2007) A genome-wide association study of global gene expression. Nat Genet 39: 1202–1207. 17873877 10.1038/ng2109

[17] Cervero J, Segura V, Macias A, Gavira JJ, Montes R, et al (2012) Atrial fibrillation in pigs induces left atrial endocardial transcriptional remodelling. Thromb Haemost 108: 742–749. 22836863 10.1160/TH12-05-0285

[18] Thijssen VL, van der Velden HM, van Ankeren EP, Ausma J, Allessie MA, et al (2002) Analysis of altered gene expression during sustained atrial fibrillation in the goat. Cardiovasc Res 54: 427–437. 12062347 10.1016/s0008-6363(02)00260-2

[19] Barth AS, Merk S, Arnoldi E, Zwermann L, Kloos P, et al (2005) Reprogramming of the human atrial transcriptome in permanent atrial fibrillation: expression of a ventricular-like genomic signature. Circ Res 96: 1022–1029. 15817885 10.1161/01.RES.0000165480.82737.33

[20] Nattel S (2002) New ideas about atrial fibrillation 50 years on. Nature 415: 219–226. 11805846 10.1038/415219a

[21] Van Gelder IC, Brundel BJ, Henning RH, Tuinenburg AE, Tieleman RG, et al (1999) Alterations in gene expression of proteins involved in the calcium handling in patients with atrial fibrillation. J Cardiovasc Electrophysiol 10: 552–560. 10355697 10.1111/j.1540-8167.1999.tb00712.x

[22] Brundel BJ, van Gelder IC, Henning RH, Tuinenburg AE, Deelman LE, et al (1999) Gene expression of proteins influencing the calcium homeostasis in patients with persistent and paroxysmal atrial fibrillation. Cardiovasc Res 42: 443–454. 10533580 10.1016/s0008-6363(99)00045-0

[23] Goette A, Staack T, Rocken C, Arndt M, Geller JC, et al (2000) Increased expression of extracellular signal-regulated kinase and angiotensin-converting enzyme in human atria during atrial fibrillation. J Am Coll Cardiol 35: 1669–1677. 10807475 10.1016/s0735-1097(00)00611-2

[24] Gao M, Wang J, Wang Z, Zhang Y, Sun H, et al (2013) An altered expression of genes involved in the regulation of ion channels in atrial myocytes is correlated with the risk of atrial fibrillation in patients with heart failure. Exp Ther Med 5: 1239–1243. 23599743 10.3892/etm.2013.949 PMC3628869

[25] Gaborit N, Steenman M, Lamirault G, Le Meur N, Le Bouter S, et al (2005) Human atrial ion channel and transporter subunit gene-expression remodeling associated with valvular heart disease and atrial fibrillation. Circulation 112: 471–481. 16027256 10.1161/CIRCULATIONAHA.104.506857

[26] Kannel WB, Feinleib M, McNamara PM, Garrison RJ, Castelli WP (1979) An investigation of coronary heart disease in families. The Framingham offspring study. Am J Epidemiol 110: 281–290. 474565 10.1093/oxfordjournals.aje.a112813

[27] Wolf PA, Abbott RD, Kannel WB (1991) Atrial fibrillation as an independent risk factor for stroke: the Framingham Study. Stroke 22: 983–988. 1866765 10.1161/01.str.22.8.983

[28] Piccini JP, Hammill BG, Sinner MF, Jensen PN, Hernandez AF, et al (2012) Incidence and prevalence of atrial fibrillation and associated mortality among Medicare beneficiaries, 1993–2007. Circ Cardiovasc Qual Outcomes 5: 85–93. 22235070 10.1161/CIRCOUTCOMES.111.962688 PMC3332107

[29] Joehanes R, Ying S, Huan T, Johnson AD, Raghavachari N, et al (2013) Gene expression signatures of coronary heart disease. Arterioscler Thromb Vasc Biol 33: 1418–1426. 23539218 10.1161/ATVBAHA.112.301169 PMC3684247

[30] Irizarry RA, Hobbs B, Collin F, Beazer-Barclay YD, Antonellis KJ, et al (2003) Exploration, normalization, and summaries of high density oligonucleotide array probe level data. Biostatistics 4: 249–264. 12925520 10.1093/biostatistics/4.2.249

[31] Alonso A, Krijthe BP, Aspelund T, Stepas KA, Pencina MJ, et al (2013) Simple risk model predicts incidence of atrial fibrillation in a racially and geographically diverse population: the CHARGE-AF consortium. J Am Heart Assoc 2: e000102. 23537808 10.1161/JAHA.112.000102 PMC3647274

[32] Benjamini Y, Hochberg Y (1995) Controlling the false discovery rate: a practical and powerful approach to multiple testing. Journal of the Royal Statistical Society, Series B (Methodological) 57: 289–300.

[33] Jia P, Zheng S, Long J, Zheng W, Zhao Z (2011) dmGWAS: dense module searching for genome-wide association studies in protein-protein interaction networks. Bioinformatics 27: 95–102. 21045073 10.1093/bioinformatics/btq615 PMC3008643

[34] Cowley MJ, Pinese M, Kassahn KS, Waddell N, Pearson JV, et al (2012) PINA v2.0: mining interactome modules. Nucleic Acids Res 40: D862–865. 22067443 10.1093/nar/gkr967 PMC3244997

[35] Ideker T, Ozier O, Schwikowski B, Siegel AF (2002) Discovering regulatory and signalling circuits in molecular interaction networks. Bioinformatics 18 Suppl 1 S233–240. 12169552 10.1093/bioinformatics/18.suppl_1.s233

[36] Chen Y, Zhu J, Lum PY, Yang X, Pinto S, et al (2008) Variations in DNA elucidate molecular networks that cause disease. Nature 452: 429–435. 18344982 10.1038/nature06757 PMC2841398

[37] Heerdt PM, Kant R, Hu Z, Kanda VA, Christini DJ, et al (2012) Transcriptomic analysis reveals atrial KCNE1 down-regulation following lung lobectomy. J Mol Cell Cardiol 53: 350–353. 22641150 10.1016/j.yjmcc.2012.05.010 PMC3418454

[38] Chang CP, Stankunas K, Shang C, Kao SC, Twu KY, et al (2008) Pbx1 functions in distinct regulatory networks to pattern the great arteries and cardiac outflow tract. Development 135: 3577–3586. 18849531 10.1242/dev.022350 PMC2680673

[39] Stankunas K, Shang C, Twu KY, Kao SC, Jenkins NA, et al (2008) Pbx/Meis deficiencies demonstrate multigenetic origins of congenital heart disease. Circ Res 103: 702–709. 18723445 10.1161/CIRCRESAHA.108.175489 PMC2633052

[40] Albritton LM, Bowcock AM, Eddy RL, Morton CC, Tseng L, et al (1992) The human cationic amino acid transporter (ATRC1): physical and genetic mapping to 13q12-q14. Genomics 12: 430–434. 1348489 10.1016/0888-7543(92)90431-q

[41] Yang Z, Venardos K, Jones E, Morris BJ, Chin-Dusting J, et al (2007) Identification of a novel polymorphism in the 3′UTR of the L-arginine transporter gene SLC7A1: contribution to hypertension and endothelial dysfunction. Circulation 115: 1269–1274. 17325243 10.1161/CIRCULATIONAHA.106.665836

[42] Yang Z, Kaye DM (2009) Mechanistic insights into the link between a polymorphism of the 3′UTR of the SLC7A1 gene and hypertension. Hum Mutat 30: 328–333. 19067360 10.1002/humu.20891

[43] Chung MK, Martin DO, Sprecher D, Wazni O, Kanderian A, et al (2001) C-reactive protein elevation in patients with atrial arrhythmias: inflammatory mechanisms and persistence of atrial fibrillation. Circulation 104: 2886–2891. 11739301 10.1161/hc4901.101760

[44] Semenza GL (2000) Expression of hypoxia-inducible factor 1: mechanisms and consequences. Biochem Pharmacol 59: 47–53. 10605934 10.1016/s0006-2952(99)00292-0

[45] Calvert JW, Cahill J, Yamaguchi-Okada M, Zhang JH (2006) Oxygen treatment after experimental hypoxia-ischemia in neonatal rats alters the expression of HIF-1alpha and its downstream target genes. J Appl Physiol 101: 853–865. 16728520 10.1152/japplphysiol.00268.2006

[46] Kido M, Du L, Sullivan CC, Li X, Deutsch R, et al (2005) Hypoxia-inducible factor 1-alpha reduces infarction and attenuates progression of cardiac dysfunction after myocardial infarction in the mouse. J Am Coll Cardiol 46: 2116–2124. 16325051 10.1016/j.jacc.2005.08.045

[47] Manalo DJ, Rowan A, Lavoie T, Natarajan L, Kelly BD, et al (2005) Transcriptional regulation of vascular endothelial cell responses to hypoxia by HIF-1. Blood 105: 659–669. 15374877 10.1182/blood-2004-07-2958

[48] Schulze-Topphoff U, Casazza S, Varrin-Doyer M, Michel K, Sobel RA, et al (2013) Tob1 plays a critical role in the activation of encephalitogenic T cells in CNS autoimmunity. J Exp Med 210: 1301–1309. 23797093 10.1084/jem.20121611 PMC3698524

[49] Bjornstad JL, Skrbic B, Marstein HS, Hasic A, Sjaastad I, et al (2012) Inhibition of SMAD2 phosphorylation preserves cardiac function during pressure overload. Cardiovasc Res 93: 100–110. 22049534 10.1093/cvr/cvr294

[50] Hyun C, Lavulo L (2006) Congenital heart diseases in small animals: part I. Genetic pathways and potential candidate genes. Vet J 171: 245–255. 16490706 10.1016/j.tvjl.2005.02.008

[51] Lin H, Dolmatova EV, Morley MP, Lunetta KL, McManus DD, et al. (2013, 10.1016/j.hrthm.2013.10.051) Gene Expression and Genetic Variation in Human Atria. Heart Rhythm. 10.1016/j.hrthm.2013.10.051 PMC3946863 24177373

[52] Adam O, Lavall D, Theobald K, Hohl M, Grube M, et al (2010) Rac1-induced connective tissue growth factor regulates connexin 43 and N-cadherin expression in atrial fibrillation. J Am Coll Cardiol 55: 469–480. 20117462 10.1016/j.jacc.2009.08.064

[53] Matkovich SJ, Zhang Y, Van Booven DJ, Dorn GW 2nd (2010) Deep mRNA sequencing for in vivo functional analysis of cardiac transcriptional regulators: application to Galphaq. Circ Res 106: 1459–1467. 20360248 10.1161/CIRCRESAHA.110.217513 PMC2891025

[54] Stranger BE, Nica AC, Forrest MS, Dimas A, Bird CP, et al (2007) Population genomics of human gene expression. Nat Genet 39: 1217–1224. 17873874 10.1038/ng2142 PMC2683249

