# Supplementary material for: Whole Blood Gene Expression and Atrial Fibrillation: The Framingham Heart Study
Source: PLoS One. 2014 May 7;9(5):e96794. doi: 10.1371/journal.pone.0096794 (PMC4013062; doi:10.1371/journal.pone.0096794)
Supplement: File S1 — Supplemental Materials. (PDF) [file pone.0096794.s001.pdf]

Whole Blood Gene Expression and Atrial Fibrillation

Table of Contents

Supplemental	Title	Page
**Table S1.**	Most significant transcripts associated with prevalent AF (FDR<0.05)	**2**
**Table S2.**	Top ten genes associated with incident AF. None of them reached the significance cutoff (FDR<0.05)	**2**
**Table S3.**	Top exons associated with prevalent AF	**3**
**Table S4.**	Association of exon expression with prevalent AF within top genes	**3-6**
**Table S5.**	Top 10 genes associated with drug treatments	**7**
**Table S6.**	Association of prevalent AF-related transcripts with types of AF during blood draw	**8**
**Table S7.**	Correlation of gene expression measured by microarray and RT-PCR	**9**
**Figure S1.**	Volcano plot of gene expression association with incident AF	**10**
	Reference	**10**

Table S1. Most significant transcripts associated with prevalent AF (FDR<0.05)

Transcript ID	Gene Symbol	Average Expression		Simple Model*			Multivariable Model +		
		AF (n=177)	No AF (n=2269)	Effect size	SE$	*P* value	Effect size	SE$	*P* value
2364677	*PBX1*	6.85	6.71	0.17	0.03	2.8×10^{-7}	0.12	0.03	7.8×10^{-4}
3712922	*C17orf39*	5.68	5.56	0.11	0.02	6.5×10^{-6}	0.07	0.02	6.4×10^{-3}
3527514	*PNP*	7.22	7.11	0.14	0.03	7.1×10^{-6}	0.10	0.04	9.3×10^{-2}
3804358	*C18orf10*	7.51	7.37	0.17	0.04	8.2×10^{-6}	0.05	0.03	1.1×10^{-2}
3507710	*SLC7A1*	5.65	5.58	0.07	0.02	1.3×10^{-5}	0.09	0.04	4.0×10^{-2}
3568534	*SPTB*	5.92	5.76	0.17	0.04	1.6×10^{-5}	0.05	0.02	3.4×10^{-3}
2849469	*ANKH*	7.34	7.26	0.10	0.02	1.9×10^{-5}	0.05	0.03	4.3×10^{-2}

$SE: Standard error
*Simple model was adjusted for age, sex and sibling relatedness
+Multivariable model was adjusted for age, sex, sibling relatedness, and additional risk factors including smoker, height, weight, systolic blood pressure, diastolic blood pressure, prevalent diabetes mellitus, prevalent myocardial infarction, prevalent heart failure and antihypertensive treatment.

Table S2. Top ten genes associated with incident AF. None of them reached the significance cutoff (FDR<0.05)

Transcript ID	Gene Symbol	Average Expression		Effect size	SE$	*P* value	FDR[31]
		AF (n=143)	No AF (n=2,126)				
3896370	*GPCPD1*	8.63	8.70	-1.60	0.37	1.5×10^{-5}	0.27
3318390	*TRIM6-TRIM34*	4.96	4.92	1.82	0.46	6.7×10^{-5}	0.60
2907459	*CNPY3*	7.66	7.70	-1.67	0.45	2.3×10^{-4}	0.99
3369249	*APIP*	6.11	6.28	-0.44	0.12	4.3×10^{-4}	0.99
2531377	*SP100*	6.80	6.74	1.00	0.29	4.4×10^{-4}	0.99
3433466	*NCRNA00173*	5.70	5.61	0.80	0.23	5.2×10^{-4}	0.99
3922100	*MX1*	7.26	7.16	0.39	0.11	5.9×10^{-4}	0.99
3362826	*LYVE1*	4.93	4.85	1.19	0.35	6.4×10^{-4}	0.99
3142217	*PAG1*	6.60	6.56	1.15	0.34	6.7×10^{-4}	0.99
3863547	*ERF*	6.31	6.35	-1.61	0.48	7.6×10^{-4}	0.99

$SE: Standard error

Table S3. Top exons associated with prevalent AF

Exon ID	Gene	Transcript	Chr	Start	End	Beta	SE	*P* value[+]
2364774	*PBX1*	NM_002585	1	164789330	164789385	0.58	0.11	$3.5x10^{-7}$
3403759	*CLEC6A*	NM_001007033	12	8618143	8618201	0.61	0.13	$4.0x10^{-6}$
3442947	*C3AR1*	NM_004054	12	8211915	8211969	0.66	0.15	$5.5x10^{-6}$
2873189	*CEP120*	NM_153223	5	122717820	122717889	-0.97	0.21	$5.6x10^{-6}$
2498994	*GCC2*	NR_028063	2	109086387	109086481	-0.72	0.16	$9.5x10^{-6}$
2505541	*PTPN18*	NM_014369	2	131117183	131117218	0.80	0.18	$9.7x10^{-6}$
3442952	*C3AR1*	NM_004054	12	8212733	8212772	0.61	0.14	$1.0x10^{-5}$
2737627	*BANK1*	NM_017935	4	102776213	102776342	-0.63	0.14	$1.1x10^{-5}$
3367826	*DCDC5*	NM_020869	11	30921903	30921941	-0.82	0.19	$1.2x10^{-5}$
3443471	*PZP*	NM_002864	12	9304212	9304256	-0.76	0.17	$1.2x10^{-5}$

Whole Blood Gene Expression and Atrial Fibrillation

Table S4. Association of exon expression with prevalent AF within top genes

Exon ID	Gene	Transcript	Chr	Start	End	Beta	SE	*P* value[+]
2849474	*ANKH*	NM_054027	5	14709296	14709358	0.33	0.25	$1.9x10^{-1}$
2849475	*ANKH*	NM_054027	5	14709524	14709912	0.32	0.28	$2.4x10^{-1}$
2849476	*ANKH*	NM_054027	5	14709939	14709971	0.05	0.16	$7.5x10^{-1}$
2849477	*ANKH*	NM_054027	5	14710169	14710492	0.42	0.23	$7.0x10^{-2}$
2849478	*ANKH*	NM_054027	5	14710702	14710736	0.06	0.20	$7.7x10^{-1}$
2849479	*ANKH*	NM_054027	5	14710831	14711039	0.44	0.24	$6.6x10^{-2}$
2849480	*ANKH*	NM_054027	5	14711151	14711273	0.38	0.20	$5.2x10^{-2}$
2849481	*ANKH*	NM_054027	5	14711344	14711386	0.20	0.17	$2.3x10^{-1}$
2849485	*ANKH*	NM_054027	5	14712983	14713044	0.26	0.15	$8.0x10^{-2}$
2849488	*ANKH*	NM_054027	5	14713654	14713774	0.20	0.18	$2.6x10^{-1}$
2849493	*ANKH*	NM_054027	5	14716834	14716932	0.34	0.15	$2.3x10^{-2}$
2849506	*ANKH*	NM_054027	5	14741946	14742018	0.38	0.22	$8.9x10^{-2}$
2849509	*ANKH*	NM_054027	5	14745989	14746070	0.24	0.19	$2.1x10^{-1}$
2849512	*ANKH*	NM_054027	5	14749312	14749385	0.14	0.17	$4.0x10^{-1}$
2849514	*ANKH*	NM_054027	5	14751197	14751296	0.33	0.19	$7.6x10^{-2}$
2849517	*ANKH*	NM_054027	5	14755979	14756053	0.34	0.20	$8.6x10^{-2}$
2849525	*ANKH*	NM_054027	5	14758598	14758662	0.60	0.35	$8.6x10^{-2}$
2849526	*ANKH*	NM_054027	5	14758670	14758707	-0.11	0.13	$4.0x10^{-1}$
2849531	*ANKH*	NM_054027	5	14769093	14769239	0.27	0.19	$1.6x10^{-1}$
2849586	*ANKH*	NM_054027	5	14871461	14871556	0.39	0.18	$2.5x10^{-2}$
2849587	*ANKH*	NM_054027	5	14871719	14871814	0.17	0.23	$4.6x10^{-1}$
3712923	*C17orf39*	NM_024052	17	17942620	17942665	0.10	0.23	$6.7x10^{-1}$
3712924	*C17orf39*	NM_024052	17	17942779	17942906	-0.45	0.24	$6.3x10^{-2}$
3712925	*C17orf39*	NM_024052	17	17943072	17943212	0.09	0.21	$6.8x10^{-1}$
3712930	*C17orf39*	NM_024052	17	17948480	17948520	-0.02	0.13	$8.8x10^{-1}$
3712931	*C17orf39*	NM_024052	17	17957459	17957524	0.09	0.14	$5.4x10^{-1}$
3712934	*C17orf39*	NM_024052	17	17962190	17962283	0.16	0.12	$1.8x10^{-1}$
3712935	*C17orf39*	NM_024052	17	17965170	17965276	0.35	0.16	$3.1x10^{-2}$
3712939	*C17orf39*	NM_024052	17	17968495	17968548	0.26	0.17	$1.3x10^{-1}$
3712940	*C17orf39*	NM_024052	17	17968559	17968595	0.30	0.18	$9.2x10^{-2}$
3712941	*C17orf39*	NM_024052	17	17968768	17969770	0.47	0.19	$1.1x10^{-2}$
3712942	*C17orf39*	NM_024052	17	17970311	17970346	0.36	0.16	$2.5x10^{-2}$
3712943	*C17orf39*	NM_024052	17	17970347	17970372	0.98	0.33	$2.8x10^{-3}$
3712944	*C17orf39*	NM_024052	17	17970394	17970514	0.53	0.17	$2.4x10^{-3}$
3712945	*C17orf39*	NM_024052	17	17970533	17970638	0.76	0.18	$2.2x10^{-5}$
3712946	*C17orf39*	NM_024052	17	17970951	17971166	0.38	0.15	$1.4x10^{-2}$
3804379	*C18orf10*	NM_015476	18	34376037	34376061	0.19	0.08	$1.7x10^{-2}$
3804380	*C18orf10*	NM_015476	18	34376387	34376700	0.33	0.14	$1.7x10^{-2}$
3804381	*C18orf10*	NM_015476	18	34376774	34376858	0.44	0.25	$7.2x10^{-2}$
3804382	*C18orf10*	NM_015476	18	34376860	34376890	0.31	0.20	$1.2x10^{-1}$
3804383	*C18orf10*	NM_015476	18	34376914	34377006	0.23	0.14	$1.0x10^{-1}$

Exon ID	Gene	Transcript	Chr	Start	End	Beta	SE	*P* value[+]
3804387	*C18orf10*	NM_015476	18	34378436	34378561	0.49	0.16	2.0×10^{-3}
3804388	*C18orf10*	NM_015476	18	34380197	34380252	0.38	0.16	1.7×10^{-2}
3804391	*C18orf10*	NM_015476	18	34385338	34385450	0.53	0.18	2.3×10^{-3}
3804392	*C18orf10*	NM_015476	18	34387825	34387876	0.43	0.13	1.3×10^{-3}
3804398	*C18orf10*	NM_015476	18	34398863	34398923	0.32	0.12	6.1×10^{-3}
3804403	*C18orf10*	NM_015476	18	34408646	34408671	0.49	0.21	2.3×10^{-2}
3804404	*C18orf10*	NM_015476	18	34408695	34408727	-0.28	0.48	5.7×10^{-1}
3804405	*C18orf10*	NM_015476	18	34408731	34408760	-0.17	0.17	3.1×10^{-1}
2364682	*PBX1*	NM_002585	1	164528976	164529054	0.43	0.40	2.8×10^{-1}
2364683	*PBX1*	NM_002585	1	164529060	164529104	0.35	0.18	6.0×10^{-2}
2364684	*PBX1*	NM_002585	1	164529213	164529246	0.39	0.14	6.0×10^{-3}
2364688	*PBX1*	NM_002585	1	164532478	164532508	0.23	0.12	6.8×10^{-2}
2364755	*PBX1*	NM_002585	1	164761731	164761823	0.56	0.18	1.4×10^{-3}
2364756	*PBX1*	NM_002585	1	164761840	164761883	-0.17	0.48	7.3×10^{-1}
2364757	*PBX1*	NM_002585	1	164761884	164761917	0.39	0.13	2.1×10^{-3}
2364758	*PBX1*	NM_002585	1	164761933	164761975	0.14	0.12	2.4×10^{-1}
2364766	*PBX1*	NM_002585	1	164768939	164769115	0.54	0.23	2.0×10^{-2}
2364770	*PBX1*	NM_002585	1	164776789	164776858	0.42	0.16	8.6×10^{-3}
2364771	*PBX1*	NM_002585	1	164781247	164781370	0.51	0.16	1.2×10^{-3}
2364774	*PBX1*	NM_002585	1	164789330	164789385	0.58	0.11	3.5×10^{-7}
2364777	*PBX1*	NM_002585	1	164790778	164790861	0.40	0.16	1.2×10^{-2}
2364796	*PBX1*	NM_002585	1	164815822	164815875	0.90	0.31	3.5×10^{-3}
2364797	*PBX1*	NM_002585	1	164815887	164815912	0.31	0.28	2.6×10^{-1}
2364798	*PBX1*	NM_002585	1	164815972	164816100	0.37	0.12	2.6×10^{-3}
2364799	*PBX1*	NM_002585	1	164816148	164816266	0.39	0.22	7.0×10^{-2}
3527518	*PNP*	NM_000270	14	20940502	20940615	0.51	0.17	2.3×10^{-3}
3527520	*PNP*	NM_000270	14	20942691	20942734	0.19	0.16	2.3×10^{-1}
3527522	*PNP*	NM_000270	14	20942940	20943068	0.53	0.17	2.0×10^{-3}
3527523	*PNP*	NM_000270	14	20943071	20943107	0.58	0.16	2.9×10^{-4}
3527525	*PNP*	NM_000270	14	20943239	20943321	0.72	0.19	2.1×10^{-4}
3527527	*PNP*	NM_000270	14	20944558	20944747	0.71	0.20	3.7×10^{-4}
3507711	*SLC7A1*	NM_003045	13	30083701	30084321	0.07	0.20	7.1×10^{-1}
3507712	*SLC7A1*	NM_003045	13	30084481	30084540	0.03	0.12	8.3×10^{-1}
3507713	*SLC7A1*	NM_003045	13	30084873	30085399	0.29	0.21	1.7×10^{-1}
3507714	*SLC7A1*	NM_003045	13	30087444	30088007	0.01	0.19	9.7×10^{-1}
3507715	*SLC7A1*	NM_003045	13	30088624	30088713	0.12	0.18	5.1×10^{-1}
3507720	*SLC7A1*	NM_003045	13	30090291	30090361	0.09	0.17	5.9×10^{-1}
3507723	*SLC7A1*	NM_003045	13	30091293	30091432	0.11	0.23	6.3×10^{-1}
3507724	*SLC7A1*	NM_003045	13	30091732	30091919	0.01	0.13	9.3×10^{-1}
3507728	*SLC7A1*	NM_003045	13	30093605	30093632	0.06	0.16	6.9×10^{-1}
3507729	*SLC7A1*	NM_003045	13	30093660	30093684	0.21	0.12	8.2×10^{-2}
3507731	*SLC7A1*	NM_003045	13	30096458	30096567	0.42	0.15	5.6×10^{-3}
3507733	*SLC7A1*	NM_003045	13	30097430	30097581	0.48	0.24	4.4×10^{-2}

Exon ID	Gene	Transcript	Chr	Start	End	Beta	SE	*P* value[+]
3507734	*SLC7A1*	NM_003045	13	30098282	30098374	0.27	0.21	$2.1x10^{-1}$
3507736	*SLC7A1*	NM_003045	13	30104675	30104718	0.66	0.17	$7.5x10^{-5}$
3507737	*SLC7A1*	NM_003045	13	30104772	30104846	0.62	0.15	$2.3x10^{-5}$
3507740	*SLC7A1*	NM_003045	13	30106975	30107119	0.29	0.16	$5.8x10^{-2}$
3507743	*SLC7A1*	NM_003045	13	30110005	30110323	0.18	0.19	$3.4x10^{-1}$
3507746	*SLC7A1*	NM_003045	13	30127900	30127989	0.30	0.21	$1.5x10^{-1}$
3507757	*SLC7A1*	NM_003045	13	30169577	30169715	0.00	0.20	1.0
3568488	*SPTB*	NM_001024858	14	65213002	65213027	-0.29	0.21	$1.7x10^{-1}$
3568489	*SPTB*	NM_001024858	14	65213042	65213211	-0.11	0.27	$6.9x10^{-1}$
3568537	*SPTB*	NM_001024858	14	65233455	65233488	0.38	0.13	$2.9x10^{-3}$
3568538	*SPTB*	NM_001024858	14	65234021	65234064	0.38	0.12	$1.9x10^{-3}$
3568539	*SPTB*	NM_001024858	14	65234417	65234567	0.43	0.12	$3.1x10^{-4}$
3568540	*SPTB*	NM_001024858	14	65235757	65235829	0.42	0.28	$1.3x10^{-1}$
3568541	*SPTB*	NM_001024858	14	65236337	65236436	0.40	0.16	$1.2x10^{-2}$
3568542	*SPTB*	NM_001024858	14	65237622	65237826	0.31	0.18	$7.5x10^{-2}$
3568543	*SPTB*	NM_001024858	14	65239402	65239604	0.26	0.15	$8.7x10^{-2}$
3568544	*SPTB*	NM_001024858	14	65239940	65240110	0.07	0.18	$7.0x10^{-1}$
3568545	*SPTB*	NM_001024858	14	65241158	65241219	0.28	0.25	$2.6x10^{-1}$
3568546	*SPTB*	NM_001024858	14	65241851	65242042	0.22	0.21	$3.0x10^{-1}$
3568547	*SPTB*	NM_001024858	14	65245880	65245951	0.01	0.16	$9.3x10^{-1}$
3568548	*SPTB*	NM_001024858	14	65246455	65246642	0.21	0.14	$1.3x10^{-1}$
3568549	*SPTB*	NM_001024858	14	65249019	65249237	0.32	0.15	$3.2x10^{-2}$
3568550	*SPTB*	NM_001024858	14	65250976	65251070	0.19	0.10	$6.2x10^{-2}$
3568551	*SPTB*	NM_001024858	14	65252264	65252344	0.21	0.12	$7.1x10^{-2}$
3568552	*SPTB*	NM_001024858	14	65252496	65252594	0.25	0.11	$2.2x10^{-2}$
3568553	*SPTB*	NM_001024858	14	65253462	65253809	0.38	0.16	$1.6x10^{-2}$
3568554	*SPTB*	NM_001024858	14	65258446	65258528	0.72	0.24	$2.9x10^{-3}$
3568555	*SPTB*	NM_001024858	14	65260123	65260375	0.31	0.14	$3.2x10^{-2}$
3568556	*SPTB*	NM_001024858	14	65261191	65261335	0.44	0.13	$8.5x10^{-4}$
3568557	*SPTB*	NM_001024858	14	65262159	65262356	0.30	0.12	$1.6x10^{-2}$
3568558	*SPTB*	NM_001024858	14	65263280	65263384	0.30	0.16	$6.1x10^{-2}$
3568559	*SPTB*	NM_001024858	14	65264449	65264531	0.17	0.12	$1.5x10^{-1}$
3568560	*SPTB*	NM_001024858	14	65266479	65266621	0.36	0.13	$6.4x10^{-3}$
3568561	*SPTB*	NM_001024858	14	65267474	65267586	0.46	0.13	$4.7x10^{-4}$
3568562	*SPTB*	NM_001024858	14	65268011	65268118	0.35	0.13	$5.7x10^{-3}$
3568563	*SPTB*	NM_001024858	14	65268472	65268497	0.26	0.11	$1.8x10^{-2}$
3568564	*SPTB*	NM_001024858	14	65268519	65268552	0.22	0.09	$1.5x10^{-2}$
3568565	*SPTB*	NM_001024858	14	65268982	65269018	0.23	0.11	$3.1x10^{-2}$
3568566	*SPTB*	NM_001024858	14	65270358	65270481	0.25	0.13	$6.3x10^{-2}$
3568567	*SPTB*	NM_001024858	14	65271716	65271784	0.24	0.12	$3.7x10^{-2}$
3568568	*SPTB*	NM_001024858	14	65289678	65289812	0.37	0.17	$2.7x10^{-2}$
3568569	*SPTB*	NM_001024858	14	65289816	65289842	-0.11	0.13	$4.2x10^{-1}$

Table S5. Top 10 genes associated with drug treatments

Warfarin		Beta blocker		Digoxin		Calcium channel blocker	
**Gene**	***P* value**	**Gene**	***P* value**	**Gene**	***P* value**	**Gene**	***P* value**
MMAA	$4.7x10^{-7}$	*RAPH1*	$7.1x10^{-16}$	*P2RX1*	$1.1x10^{-5}$	*TSPAN2*	$5.0x10^{-15}$
RAP2A	$1.7x10^{-6}$	*CX3CR1*	$6.1x10^{-15}$	*PTP4A3*	$1.5x10^{-5}$	*TSC22D3*	$1.1x10^{-13}$
RAP1GAP	$2.5x10^{-6}$	*ADRB2*	$1.3x10^{-12}$	*GPR112*	$2.3x10^{-5}$	*MYADM*	$1.4x10^{-13}$
SLC7A1	$1.9x10^{-5}$	*HIF1A*	$1.3x10^{-12}$	*KIR3DX1*	$3.9x10^{-5}$	*ANKRD28*	$9.7x10^{-12}$
METTL9	$1.9x10^{-5}$	*EPS8*	$2.4x10^{-12}$	*MAGI3*	$4.4x10^{-5}$	*FAR2*	$1.9x10^{-11}$
TSTA3	$2.6x10^{-5}$	*UBE2J1*	$6.3x10^{-12}$	*SULT2A1*	$5.3x10^{-5}$	*IDS*	$9.1x10^{-11}$
IFITM1	$2.9x10^{-5}$	*CDC42EP3*	$2.4x10^{-11}$	*PPP2R5D*	$9.4x10^{-5}$	*ARHGEF40*	$4.4x10^{-10}$
ATG14	$4.2x10^{-5}$	*TLR2*	$2.6x10^{-11}$	*CHRNB2*	$1.0x10^{-4}$	*RFX2*	$1.3x10^{-9}$
CUL4A	$5.4x10^{-5}$	*KYNU*	$7.0x10^{-11}$	*FAM123A*	$1.5x10^{-4}$	*SLC31A2*	$3.0x10^{-9}$
LOC285902	$1.1x10^{-4}$	*PDE4B*	$3.6x10^{-10}$	*FGF6*	$1.6x10^{-4}$	*FOS*	$7.3x10^{-9}$

Table S6. Association of prevalent AF-related transcripts with types of AF during blood draw

Transcript ID	Gene Symbol	Sinus rhythm during blood draw (n=115)			AF during blood draw (n=62)			All prevalent AF (n=177)		
		Effect size	SE*	*P* value	Effect size	SE*	*P* value	Effect size	SE*	*P* value
2364677	*PBX1*	0.15	0.04	$2.0x10^{-4}$	0.21	0.05	$1.1x10^{-4}$	0.17	0.03	$2.8x10^{-7}$
3712922	*C17orf39*	0.09	0.03	$1.6x10^{-3}$	0.14	0.04	$3.8x10^{-4}$	0.11	0.02	$6.5x10^{-6}$
3527514	*PNP*	0.11	0.04	$2.5x10^{-3}$	0.18	0.05	$2.1x10^{-4}$	0.14	0.03	$7.1x10^{-6}$
3804358	*C18orf10*	0.16	0.05	$3.4x10^{-4}$	0.17	0.06	$4.2x10^{-3}$	0.17	0.04	$8.2x10^{-6}$
3507710	*SLC7A1*	0.05	0.02	$4.4x10^{-3}$	0.10	0.03	$9.2x10^{-5}$	0.07	0.02	$1.3x10^{-5}$
3568534	*SPTB*	0.13	0.05	$6.1x10^{-3}$	0.24	0.06	$1.4x10^{-4}$	0.17	0.04	$1.6x10^{-5}$
2849469	*ANKH*	0.09	0.03	$1.3x10^{-3}$	0.12	0.04	$1.4x10^{-3}$	0.10	0.02	$1.9x10^{-5}$

*SE: Standard error

Table S7. Correlation of gene expression measured by microarray and RT-PCR

Gene	Pearson's correlation coefficient	Gene	Pearson's correlation coefficient	Gene	Pearson's correlation coefficient	Gene	Pearson's correlation coefficient
ABCA6	0.22	*DPCD*	0.62	*MAOA*	0.41	*SLC25A37*	0.65
ABCA9	0.26	*EGR2*	0.29	*MKRN1*	0.51	*SLC25A39*	0.73
ABCC13	0.21	*EPB49*	0.09	*MYL4*	0.73	*SLC4A1*	0.64
ACTB	0.00	*FAM188A*	-0.07	*NEB*	0.21	*SLC6A16*	0.18
ADIPOR1	0.66	*FBXL3*	0.14	*NOTCH2*	0.61	*SLC7A11*	0.30
AHSP	0.89	*FIS1*	0.34	*NOX4*	0.01	*SLPI*	0.65
ALAS2	0.39	*FKBP8*	0.70	*OAT*	0.28	*SPATS2L*	0.39
ANGPTL1	-0.01	*GABARAPL2*	0.71	*OLFM1*	0.21	*SPTA1*	0.43
B2M	0.16	*GARS*	0.08	*OSBPL10*	0.23	*SPTB*	0.04
BPGM	0.67	*GUK1*	0.74	*PCTP*	0.65	*SRRD*	0.50
BSG	0.77	*GYPA*	0.61	*PDCD1LG2*	0.30	*SUPV3L1*	0.17
CA1	0.79	*GYPB*	0.74	*PHEX*	0.24	*TESC*	0.79
CALCRL	0.20	*GYPC*	0.67	*PI3*	0.81	*TFDP1*	0.60
CCDC144A	0.01	*GYPE*	0.04	*PRR13*	0.01	*TMEM56*	0.52
CCNDBP1	0.65	*GZMB*	0.45	*PSMD12*	0.17	*TMTC2*	0.39
CDC34	0.71	*HAGH*	0.75	*RBM38*	0.75	*TSPAN5*	0.82
CHPT1	0.48	*HBD*	0.82	*RPA2*	0.24	*TSTA3*	0.66
CISD2	0.67	*HBM*	0.73	*RPIA*	0.64	*UBXN6*	0.73
CLC	0.54	*HBQ1*	0.53	*RPL41*	0.10	*XK*	0.61
CRADD	0.14	*IL23A*	0.16	*SCARB2*	0.16	*ZDHHC2*	0.54
CREG1	0.51	*INPP5F*	0.27	*SELENBP1*	0.52	*ZNF195*	0.07
CTNNAL1	0.49	*ISCU*	0.57	*SGIP1*	0.08	*ZNF25*	0.10
CXCL9	0.13	*LAMP3*	0.39	*SIAH2*	0.61	*ZNF800*	-0.09
DLEU2	-0.05	*LCP2*	0.51	*SLC14A1*	0.80		

Figure S1. Volcano plot of gene expression association with incident AF

Each dot represents one gene. The x-axis represents the beta of each gene, whereas the y-axis represents the $\log_{10}$(*P* value). Positive beta represents that the gene expression was associated with increased hazard of incident AF, whereas negative betas represent that the gene expression was associated with decreased hazard of incident AF. No gene reached the significant cutoff (FDR<0.05).

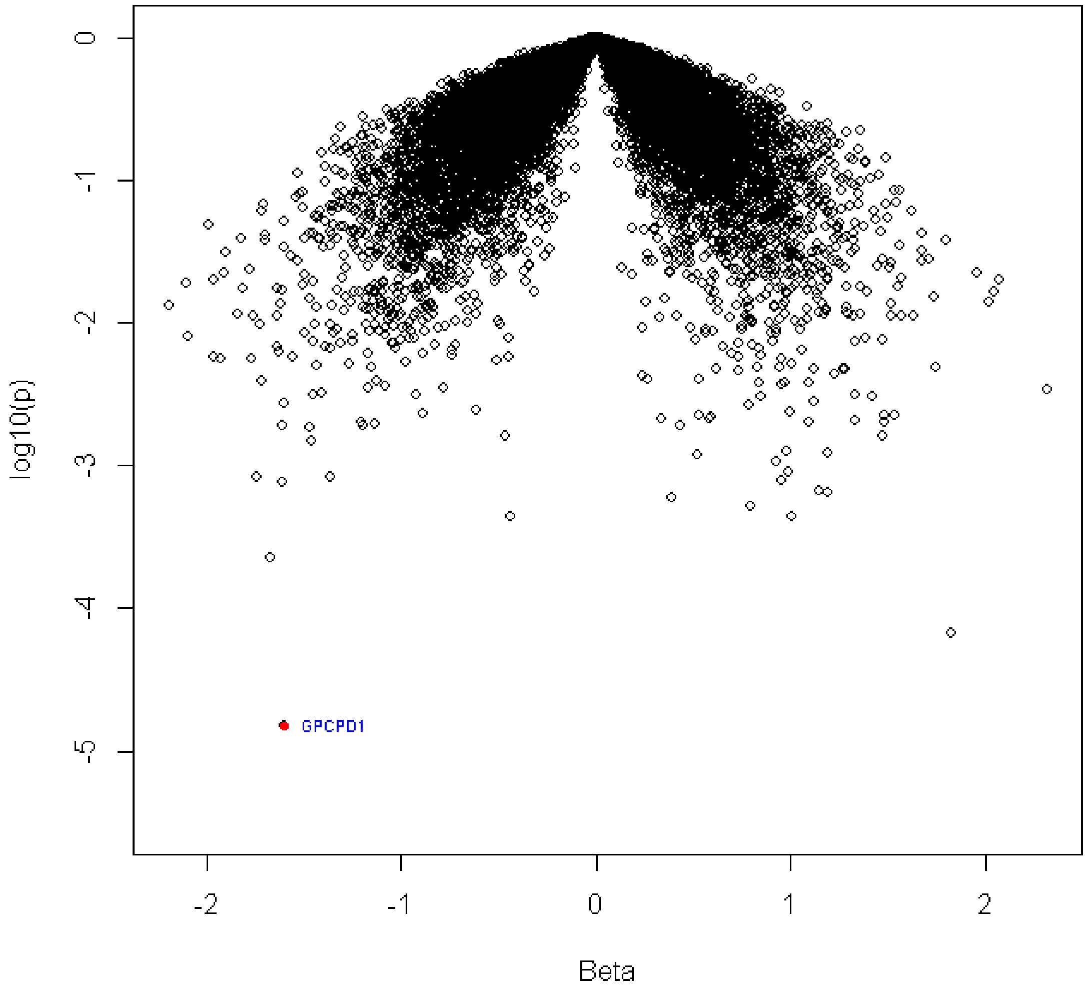

References

1. Ellinor PT, Lunetta KL, Albert CM, Glazer NL, Ritchie MD, Smith AV, et al. Meta-analysis identifies six new susceptibility loci for atrial fibrillation. *Nat Genet*. 2012;44:670-675
